# Creating a Data Cleaning and Pre-Processing Module for Generalisable Data Linkage

**DOI:** 10.23889/ijpds.v9i5.2866

**Published:** 2024-09-18

**Authors:** Josie Plachta, Mary Cleaton, Leah Quinn, Alex Mackay, Zoe White

**Affiliations:** 1 Office for National Statistics

## Objective

The Office for National Statistics (ONS) are developing a generalisable tool to facilitate the linkage of various datasets to its population-spine. However, a generalisable process requires that a variety of input datasets can be adaptively pre-processed – which is a problem for bespoke methodologies. Key requirements of the cleaning pipeline include minimal input from the user, and scalability and efficiency to work on Big Data.

## Approach

The pipeline must recognise and adjust the pre-processing steps applied based on the variables present and user requirements. It must accept, preprocess, and derive consistent standardised variables from a variety of input variables and formats, including complex data characteristics.

## Results

The MVP pipeline successfully met the requirements. It is based on a three-level hierarchy of functions, allowing flexibility and complexity in data preparation. With minimal user input, a variety of important linkage variables are cleaned, and additional variables derived consistently.

## Conclusions

The module has shown promising results at scale, successfully pre-processing datasets of over 91 million records. It will be a valuable tool for increasing the ease and efficiency of record linkage to the ONS’ population-spine. This will make linked data more accessible and increase the consistency of linked datasets, improving compatibility for onward linkages and the comparability of results.

Future work will involve applying this cleaning method to a wider range of different datasets to further test the generalisability of the method, and increasing the adaptability of the module to allow for even greater variation in the input datasets.

